# Value of the Dental Society

**Published:** 1906-06-15

**Authors:** R. W. Morse

**Affiliations:** Lansing, Mich.


### VALUE OF THE DENTAL SOCIETY.

BY R. W. MORSE, D.D.S., LANSING, MICH.

Address before the Central Michigan Dental Society, February, 1906.

As President of the Central Michigan Dental Society and citizen of Lansing, I cordially welcome you to our city, and this the fifth session of our society. Previous meetings have been satisfactory in regard to attendance and enthusiasm. The papers presented have been of interest and excellence, and the benefit to the members has been greater than many would expect. Hence we are justified in continuing the work of enlarging our society and widening its horizon of endeavor.

This session of our society shows the progressive spirit of the profession in this part of Michigan. Each one in attendance has laid aside his routine office duties for a conference with his fellow-workers, with the object of improving his efficiency in the work he does for the people who entrust to his care the repair and protection of their teeth.

Many methods of doing the same kind of work are advocated, and while experience is a good teacher, shs is slow and tedious in eliminating poor methods, but by comparison, we can more readily advance toward perfection. We are looking for improvement in methods, appliances and materials, many of which have been assembled here for our benefit.

One of the indications of the advancement of dental art is reflected by the esthetic demands of our patients. They realize the horror of the obstrusive gold crown and are requiring that the restoration of tooth structure be the least conspicuous possible, also the fact that they have become educated to realize teeth were made for service and not to be extracted is another credit mark for our profession.

To the dentist who attends this society, these things have been repeatedly emphasized and by passing them on to his patients and working up to the principle of tooth saving with the least disfigurement, our profession will make progress of the right sort, and we shall receive the confidence of appreciative patients. It is in this way that the ethical practitioner gains business success and at the same time frees himself from the drudgery of a perfunctory service.

Last year it was voted to hold the meetings of the society in the future in Lansing on account of its accessibility and central location. We believe this to be an excellent plan and hope you will all take a personal interest and pride in making these meetings interesting and successful. This you can do by bringing some of your work to be given as a clinic, and every man can show his method of doing something in a manner that will be instructive and of interest to some one.

It is difficult to get enough clinics to satisfy everybody and I would ask each one present at this meeting to have at least one ready to demonstrate at our next meeting, and when the first notice of the meeting is sent out, write to the Secretary and tell him the kind of a clinic you will give. This he will appreciate and so will the society.

Every dentist owes it to himself and his patrons to put himself in position to best administer to their wants. The Central Michigan Society contributes to that end. Each member should contribute to making the meeting a success and therefore should do something, and you are equal to the occasion if you will try.

We have a good program of papers and clinics for this session and I trust everyone may feel free to engage in the discussion and give close attention to the clinics.

